# C-Reactive Protein Is Elevated Only in High Creatine Kinase Responders to Muscle Damaging Exercise

**DOI:** 10.3389/fphys.2019.00086

**Published:** 2019-02-11

**Authors:** Ashwin W. Isaacs, Filippo Macaluso, Carine Smith, Kathryn H. Myburgh

**Affiliations:** ^1^Department of Physiological Sciences, Stellenbosch University, Stellenbosch, South Africa; ^2^Department of Experimental Biomedicine and Clinical Neurosciences, University of Palermo, Palermo, Italy; ^3^Euro-Mediterranean Institute of Science and Technology, Palermo, Italy; ^4^SMART Engineering Solutions & Technologies Research Center, eCampus University, Novedrate, Italy

**Keywords:** eccentric exercise and muscle damage, creatine kinase, myoglobin, single nucleotide polymorphism, myeloperoxidase

## Abstract

The purpose of this study was to investigate if exertional rhabdomyolysis induced by an acute bout of plyometric exercise in untrained individuals was associated with histological characteristics of skeletal muscle, creatine kinase (CK) polymorphism or secondary damage. Twenty-six healthy male untrained individuals completed a bout of plyometric exercise (10 sets of 10 maximal squat jumps, with each standardized to achieve at least 95% of individual maximal jump height). Blood samples were taken, and perceived pain was scored immediately before the exercise intervention and 6 h, 1, 2, and 3 days post-intervention. Muscle biopsies were collected 9 or 4 days before (baseline) and 3 days after plyometric jumps. Subjects were divided into two groups, high (*n* = 10) and low responders (*n* = 16), based on a cut-off limit for exertional rhabdomyolysis of peak CK activity ≥ 1000 U/L in any post-exercise blood sample. Perceived pain was more severe assessed in squat than standing position. Low responders perceived more pain at 6 h and 1 day, while high responders perceived more pain than low responders on days three and four after exercise; structural (dystrophin staining) and ultra-structural (transmission electron microscopy) analysis of muscle fibers revealed no baseline pathology; damage was evident in all individuals in both groups, with no difference between high and low responders in either damage or fiber type proportion. High responders had significantly higher total white blood cell and neutrophil counts 6 h and significantly higher C-reactive protein (CRP) 6 h and days one and two after exercise compared to low responders. High responders had significantly greater muscle myeloperoxidase (MPO) levels in baseline and 3 day post-exercise biopsies compared to baseline of low responders. MLCK C49T single polymorphism was present in 26% of volunteers, whose CK responses were not higher than those with MLCK CC or CT genotype. In conclusion, perceived pain is more effectively assessed with potentially affected muscle under eccentric strain, even if static. High CK responders also have pronounced CRP responses to unaccustomed plyometric exercise intervention. Exertional rhabdomyolysis after unaccustomed eccentric exercise may be related to underlying inability to resolve intramuscular MPO.

## Introduction

Elevated circulating creatine kinase (CK) is a hallmark of muscle damage caused by intense and unaccustomed exercise including muscle-group targeted eccentric resistance exercise, downhill running and plyometric jumping (Nosaka et al., 2001; Macaluso et al., 2012b, 2013; van de Vyver and Myburgh, 2012). Literature reports that certain individuals, despite apparently similar characteristics to other study participants, may experience greater increases in CK activity following unaccustomed eccentric exercise (Clarkson et al., 2005; Devaney et al., 2007; Yamin et al., 2008). This biological phenomenon remains unexplained (Sayers and Clarkson, 2002).

Individuals who experience severe muscle damage may present with exertional rhabdomyolysis which is characterized by the continued release of myoglobin (Mb) into the circulation (Sayers and Clarkson, 2002; Scalco et al., 2016). Typically, elevations in CK and Mb go hand and in hand after extreme bouts of unaccustomed eccentric exercise (Clarkson et al., 2005), but CK activity is most commonly measured (Damas et al., 2016). The laboratory diagnostic cut-off limit for exertional rhabdomyolysis varies across studies and study populations (Sayers and Clarkson, 2002; Warren et al., 2002; Lauritzen et al., 2009), however, a lower limit for exertional rhabdomyolysis has been suggested to be CK ≥ 1000 U/L (Lee and Clarkson, 2003; Thoenes, 2010). Although this phenomenon is well described, including the potential for clinical sequelae (Hill et al., 2017), the potential mechanisms pre-disposing certain individuals remains to be elucidated fully.

Potential role players could include background genetic polymorphisms, the pre-exercise condition of skeletal muscle itself or pre-exercise systemic profile – such as relative leukocyte distribution or inflammatory processes. Several genetic polymorphisms of skeletal muscle proteins have been associated with exertional muscle damage variability. A few studies have indicated that genes involved in muscle structure (ACTN3) (Clarkson et al., 2005), growth (IGF2) (Devaney et al., 2007) or regulation of force production (MYLK) (Clarkson et al., 2005), can present polymorphisms that affect baseline CK activity and exacerbate the muscle damage response to eccentric exercise. Similarly, inflammation-related polymorphisms (TNFA and IL6) have been associated with elevated CK activity following unaccustomed eccentric exercise (Yamin et al., 2008). For a recent review, see Baumert et al. (2016).

Muscle damage results in the activation and mobilization of circulating leukocytes to the damaged area, where they release reactive oxygen species (ROS) and proteolytic enzymes (Smith et al., 2008). Oxidative stress has been shown to play an important role in muscle damage (Lee and Clarkson, 2003; Yamin et al., 2008) and redox individuality has been implicated in damage variability observed after eccentric exercise (Margaritelis et al., 2014). More specifically, neutrophils are early inflammatory responders and known to be a source of oxidative stress (Vollaard et al., 2005; Smith et al., 2008). In addition, myeloperoxidase (MPO), which is also highly expressed in neutrophils and released during the respiratory burst (Childs et al., 2001), is not an oxygen radical *per se*, but at elevated concentrations is also known to cause tissue damage. Schneider and Tiidus (2007) suggested inclusion of intramuscular MPO assessment in investigations aimed at elucidating the extent of neutrophil activity in muscle (Schneider and Tiidus, 2007). Chronologically, in response to mechanical damage and neutrophil-induced secondary damage, C-reactive protein (CRP) dependent complement activation facilitates removal of cellular debris (Pereira Panza et al., 2015). CRP elevation after an acute bout of eccentric biceps exercise has been shown to be sensitive to a nutritional intervention in the form of a supplement containing antioxidants and docohexanoic acid (Phillips et al., 2003). The supplement had no effect on CK levels. This suggests that CRP should be investigated in more detail as a biomarker of secondary damage following unaccustomed exercise that may induce muscle damage.

Unaccustomed eccentric exercise damages type II muscle fibers in greater proportion than type I fibers (Macaluso et al., 2012b). Therefore, sedentary individuals with a larger percentage of type II muscle fibers may present with higher values of the indirect markers used to describe exertional muscle damage. However, whether participants with higher proportions of Type II fibers also present with higher CK activity responses, within the range of exertional rhabdomyolysis, is currently not known.

The aim of this study was to probe in untrained individuals different mechanisms which may predispose to exertional rhabdomyolysis, induced by an acute bout of plyometric exercise that results in varying levels of post-exercise circulating CK. Variables assessed were selected from different categories of potential contributors namely genetic background (MYLCK genotype), skeletal muscle phenotype (fiber type; MPO content) and inflammatory profile (circulating leukocytes; CRP).

## Materials and Methods

### Study Design

The study was granted ethical clearance (reference no. N09/05/164) by Sub-committee C of the Research Committee of University of Stellenbosch. On the day of the plyometric exercise intervention, blood samples and perceived pain scores were collected both immediately before (day 0) and 6 h 1, 2, 3 and 4 days after exercise intervention; whilst muscle biopsies were obtained 4 or 9 days before and 3 days after the acute bout of plyometric exercise.

### Subjects

Twenty-six healthy male untrained individuals (age = 21.5 ± 1.6 years; height = 175.9 ± 8.9 cm; weight = 73.0 ± 14.1 kg) were recruited. Participants were informed of the criteria for inclusion and exclusion and all the experimental procedures and associated risk before providing written informed consent. All participants completed a habitual and recent physical activity and health history questionnaire, and they were healthy and had no medical contraindications or any muscle or lower limb injury in the previous 6 months. No participants were currently or chronically treated by any corticosteroid-containing medication (including inhaled forms). Exercise criteria for inclusion were: habitually physically active 0–2 times per week without any systematic training of the lower body; no plyometric exercise of the lower body other than typical daily activities. All participants refrained from unaccustomed exercise or vigorous physical activity during the study. Volunteers were informed to maintain their normal dietary habits, and not to take any anti-inflammatory drugs (e.g., non-steroidal anti-inflammatory drugs) or nutritional supplements (e.g., vitamins, protein/amino acids) during the experimental period. The habitual exercise and healthy history questionnaire confirmed that none of the participants practiced plyometric exercise before the study.

### Plyometric Exercise Intervention

Volunteer subjects first completed 5 min of backward and forward running together with light stretching as a warm-up before performing three maximal squat jumps. The maximal height reached by the head was recorded and 95% of this height served as a target height which subjects had to maintain during the exercise intervention. The plyometric exercise intervention consisted of 10 sets of 10 maximal squat jumps, separated by 1 min recovery time between sets. This protocol has previously been used to induce transient muscle damage in the knee extensor muscles (Vissing et al., 2008; Macaluso et al., 2012b). Subjects were allowed to swing their arms which assisted with balance and generating momentum with each jump. However, by not adopting a 90° knee joint angle on landing and not maintaining an upright trunk position and the minimum jump height all constituted incorrect jumps. The jump technique was observed by the researcher and when incorrect jumps were observed, the subjects were instructed to stop and given a 1 min rest period before completing that set, thereby completing 100 proper squat jumps (Macaluso et al., 2012b). Static stretching for less than 60 s was permitted since data in literature reported that this would not affect maximal muscle performance (Kay and Blazevich, 2012).

### Blood Sampling and Analysis

After an equilibration time of 5 min in a supine position, blood samples were drawn from the antecubital vein and collected in SST and EDTA vacutainer tubes (Becton Dickinson and Company). Tubes were inverted 5–6 times, and then centrifuged at 3500 rpm for 10 min at 4°C. Blood samples were then analyzed by commercial laboratory PathCare pathology laboratory (Stellenbosch Medi Clinic, South Africa) for CK activity (Access one-step sandwich assay CK-MB assay), Mb (Mb-chemiluminescence), CRP (CRP-immunoturbidimetric method Beckman Coulter, Inc.,), total and differential white blood cell count (WBC) using a CellDyne 3700CS Hematology Analyzer (Abbott Diagnostics, Fullerton, CA, United States).

### High and Low Responders Groups

Subjects were categorized as high (*n* = 10) or low (*n* = 16) responders based on CK activity: individuals for whom CK activity at all time points assessed was lower than 1000 U/L, were considered low responders, whereas individuals exhibiting CK activity in access of 1000 U/L at any time point, were categorized as high responders. In literature CK ≥ 1000 U/L has been suggested to be lower limit for exertional rhabdomyolysis (Lee and Clarkson, 2003; Thoenes, 2010).

From this point forth the groups will be defined as high (height = 180 ± 0.05 cm; weight = 71.1 ± 10.05 kg; 95% jump height 211 ± 0.06 cm) and low responders (height = 180 ± 0.11 cm; weight = 74.2 ± 16.37 kg; 95% jump height 211 ± 0.14 cm).

### Perceived Pain

Perceived pain was measured using a visual pain scale. Participants indicated soreness of the knee-extensors ranging from 0, None; 2, Discomfort; 4, Annoying; 6, Horrible; 8, Dreadful; 10, Agonizing. This was done in two different positions (standing and squatting position) as described by Macaluso et al. (2012b).

### Muscle Sampling

Muscle biopsies of *vastus lateralis* muscle were obtained 9 or 4 days prior and 3 days after the plyometric exercise intervention using the suction-assisted technique (Macaluso et al., 2012a). Biopsies were taken by a medical doctor experienced in the technique and experienced in obtaining follow-up biopsies from the same depth. The second biopsy was taken from the opposite leg. Each biopsy was split into three parts: one was snap frozen in liquid nitrogen, the second was embedded in tissue freezing medium and frozen in isopentane (cooled in liquid nitrogen) for subsequent cryosectioning and immunofluorescent microscopy, and the third (1 × 3 mm) was fixed in 2.5% glutaraldehyde.

### Immunofluorescence Staining

Cross sections of the muscle tissue were cut using a cryostat microtome (Leica CM1100, Leica Microsystem Nussloch GmbH, Germany) at -22°C, mounted on slides and stored at -20°C. The next day the slides were brought to room temperature, rinsed in 0.01 M phosphate buffered saline (PBS) containing 0.25% Triton X-100 (15 min) and washed with PBS (3 × 5 min). The following primary antibodies were used: MHC II (1:250; A4.74, mouse monoclonal antibody, Developmental Studies Hybridoma Bank, Iowa City, IA, United States) to identify fast twitch muscle fibers and dystrophin (1:250, rabbit polyclonal, Santa Cruz Biotechnology, Santa Cruz, CA, United States) to identify the sarcolemma. The first primary antibody was left to incubate for 1 h at room temperature, after which sections were revealed with Alexa fluor 488 conjugated secondary antibody (1:250, goat anti-mouse, Invitrogen, Eugene, OR, United States) and Alexa fluor 594 (1:250, goat anti-rabbit, Invitrogen, Eugene) conjugated secondary antibody for 1 h at room temperature. bisBenzimide H 33342 trihydrochloride (Hoechst, 1:200; Sigma Aldrich, B2261) was added to all sections to visualize nuclei. Samples were then washed in PBS (3× 5 min), mounted with fluorescent mounting medium (DAKO; GLostrup, Denmark) and analyzed with a direct fluorescent microscope (model DM 5000 CTR; Leica Microsystem Nussloch GmbH) with 10× and 20× objectives. Images obtained were assessed for fiber positivity to MHC II antibody for fiber type determination (MHC-IIa fibers are highly positive; MHC IIx fibers are positive with lower intensity; MHC-I fibers are negative) and loss of dystrophin staining to determine which were damaged (Macaluso et al., 2012b, 2014).

### Transmission Electron Microscopy Analysis

Muscle specimens from each volunteer for both time points were cut in a longitudinal orientation, fixed in 2.5% glutaraldehyde and post-fixed in 1% osmium tetroxide for 1 h. Next the samples were dehydrated with graded ethanol 30, 50, 70, 95, and 100% v∖v and tissue pieces were placed in propylene oxide for 30 min and infiltrated with resin (EPON 812, Electron Microscopy Sciences, Hatfield, PA, United States) at passages of 1:3, 2:2 and 3:1 resin to propylene oxide) 1 day before polymerization at 50°C for 48 h. Ultrathin (50–70 nm) longitudinal skeletal muscle sections were cut with an ultramicrotome (model RM2125 RT; Leica Microsytem Nussloch GmbH, Germany). Images of ultrathin sections of resin-embedded muscle were obtained using a transmission electron microscope (Jeol-Jem 1011 TEM, Leica Microsystem Nussloch GmbH, Germany). The longitudinal sections were assessed for the presence of damaged sarcomeres (*Z*-disk streaming) or necrotic and hypercontracted areas as described by Lauritzen et al. (2009).

### MPO Enzyme-Linked Immunosorbent Assay

The concentration of secreted MPO in muscle tissue was determined using the Abcam MPO Human ELISA kit (ab119605), as recommended by the manufacturer. Briefly, 2 mm frozen muscle tissue was washed 2× in Phosphate-buffered saline (PBS) and homogenized in RIPA (pH 7.4, Tris–HCL 2.5 mM, EDTA 1 mM, NaF 50 mM, dithiothreitol 1 mM, phenylmethylsulfonyl fluoride (PMSF) 0.1 mM, benzamidine 1 mM, 4 mg/ml SBTI, 10 mg/ml leupeptin, 1% NP40, 0.1% SDS and 0.5% Na deoxycholate) using a tissue homogenizer (Ultra-Turrax, Germany). The homogenate was then centrifuged at 5000 × *g* for 5 min and immediately assayed. The sample readings were performed using the ELx800 universal microplate reader (Biotek Instruments, Inc., VT, United States).

### Genotyping Analysis

Snap frozen muscle tissue (baseline muscle biopsy) was sent to the Central Analytical Facility (CAF, Stellenbosch University, South Africa) to test possible single nucleotide polymorphism (SNP) MYLCK C49T. DNA extraction was performed as follows: Tissue was placed in 1.7 ml tube and homogenized with the TissueLyzer (Qiagen). Next 400 μl of Buffer PL2 (NucleoSpin Plant II Kit, Separations) with 2 μl of proteinase K (10mg/ml; Sigma-Aldrich) was added. The sample was incubated overnight in a waterbath at 60°C, following the protocol of the manufacturer. Extraction was performed on the Tecan TMP 2000 Liquid Handling Platform. The DNA extract was then analyzed for selected SNPs within the genes of selected muscle structure proteins. TaqMan primer sets for SNPs were: TTC AGA GCA ACT TCA GGA GCTT (forward primer); GCC AGT GGG ACA GGA AAGG (Reverse Primer). The PCR was performed in a cycler (Verity, Applied Biosystems, Life Technologies, CA, United States) with the following cycling conditions: 95°C for 10 min followed by 44 cycles at 92°C for 15 s and a final extension of 60°C for 1 min. Post-PCR purification was done using the NucleoFast purification system (NucleoFast, Separations, Germany). Sequencing was performed with BigDye Terminator (V1.3, Applied Biosystems, Life Technologies, CA, United States) followed by electrophoresis on the DNA Analyser (3730xl, Applied Biosystems, Life Technologies, CA, United States). Sequences were analyzed and trimmed using Sequencing Analysis (V5.3.1, Applied Biosystems, Life Technologies, CA, United States). Alignments were done using the ClustalW module, BioEdit version 7.0.4.1 with the downloaded SND-ID as reference. Possible polymorphism variants included MYLCK C49T genotype CC, CT, and TT (Clarkson et al., 2005).

### Statistical Analysis

Data were assessed for normality by inspecting normal probability plots. A logarithmic transformation (Log10) was applied to non-normally distributed data (CK, Mb, CRP, and perceived pain) before analysis. Changes in perceived pain (in squat and in standing position), blood parameters (CK, Mb, and CRP), total and differential WBC in high responders vs. low responders over time were analyzed by two-way mixed model repeated-measures analysis of variance. If a significant difference was detected, this was further evaluated by the *post hoc* Fisher’s LSD test. Statistical analyses were performed using PASW (version 18; SPSS Inc., Chicago, IL, United States). Significance was accepted at *P* ≤ 0.05.

## Results

### Muscle Damage

In the standing position, perceived pain scores for both low and high CK responders were fairly low and followed a similar time course, with significant elevations reported at 6 h and days 1 and 2 following plyometric exercise (Table 1). In contrast, in the squat position, both groups reported moderate to severe soreness, with highest scores on days 1 and 2 post-exercise, although on days 3 and 4, soreness scores were still significantly elevated from baseline in both groups. Time-dependent group differences were evident: while perceived soreness scores were higher in low responders at 6 h post-plyometric exercise, high responders rated their soreness at days 3 and 4 higher than low responders, but only in the squat position. At these later time points, the high responders’ perceived pain scores were still at least 4-fold higher than the 6 h time point, whilst that of low responders was only ≈1.5-fold higher than 6 h.

**Table 1:** Perceived muscle soreness over time before and after a single bout of plyometric exercise.

	Low responders		High responders	
Days	Standing position	Squat position	Standing position	Squat position
-4 day	0.0 ± 0.0	1.4 ± 1,6	0.0 ± 0.0	0.9 ± 1.1
0 day	0.0 ± 0.0	1.0 ± 1.2	0.0 ± 0.0	1.1 ± 1.2
6 h	1.3 ± 1.4^∗^	2.0 ± 1.0^∗^	0.9 ± 1.0^∗^ψ	1.0 ± 1.0ψ
1 day	1.5 ± 1.6^∗^	5.8 ± 1.7^∗^	1.4 ± 1.0^∗^‡	6.6 ± 1.2^∗^#
2 days	1.5 ± 2.4^∗^	6.3 ± 2.5^∗^	0.9 ± 0.9^∗^	7.5 ± 1.7^∗^#
3 days	0.60 ± 1.0	3.8 ± 1.7^∗^	0.6 ± 1.0	5.0 ± 1.0^∗^ψ
4 days	0.5 ± 1.2	2.8 ± 1.5^∗^	0.2 ± 0.4	4.4 ± 1.2^∗^ψ


*Soreness in the squat position and in the standing position for the low (n = 16) and high (n = 10) responders, respectively. ^∗^Significantly different from time points -4 and 0 day, (P < 0.0001), #significantly different from time point 6 h (P < 0.001), ‡significantly different from time point 3 and 4 days (P < 0.05), ψsignificantly different response between high and low responders (P < 0.05). Data are expressed as mean ± SD.*

**FIGURE 1:**
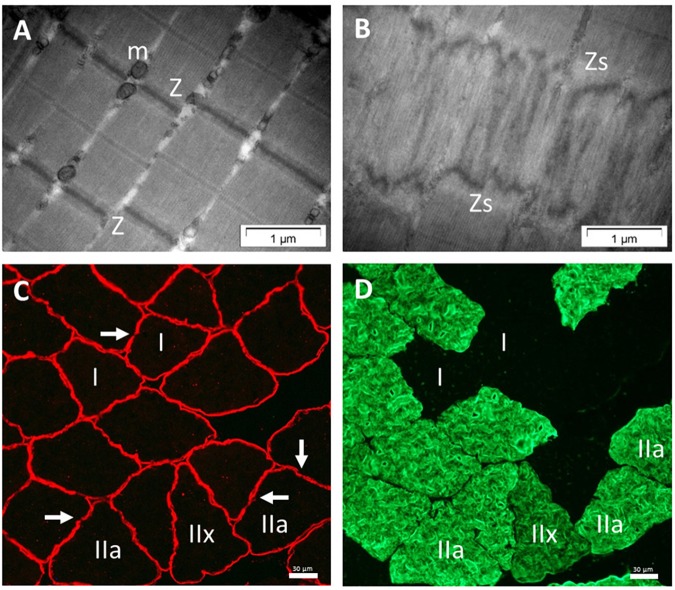
Analysis of skeletal muscle sections. Electron micrographs of longitudinal sections. Illustration of undamaged sarcomeres **(A)**. Arrangement of one sarcomere: Z, Z-line; and m, mitochondria. Damaged sarcomeres **(B)** on day 3 following eccentric exercise in human skeletal muscle: Zs, z-line streaming. No difference in ultrastructural damage was observed between low and high responder groups. Immunofluorescence of muscle cross-sections also on day 3 after the plyometric exercise intervention, using double immunostaining with anti-dystrophin **(C)** and anti-myosin heavy chain II **(D)**. Arrows show examples of the loss in dystrophin staining; IIa and IIx indicate examples of MHC-II positive fibers; I, MHC-I fibers.

Electron micrographs of muscle biopsies performed on the 3rd day after the acute bout of plyometric exercise revealed muscle damage in the form of *Z*-disk streaming in all subjects (refer to Figure 1A,B for representative images of pre- and post-exercise sections in the longitudinal plane of *vastus lateralis* muscle). Necrotic and hyper-contracted areas were not evident in any of the muscle samples. Qualitative assessment of ultrastructural muscle damage did not reveal any overt differences between high and low responders.

In line with the qualitative assessments of EM images, qualitative assessment of sarcolemmal damage, as assessed by loss of dystrophin continuity in membranes (Figure 1C) in the red channel of the double-stained sections, revealed breaks in dystrophin in both groups. Fiber type distribution did not differ between low and high responders (Table 2). Quantitative data of sarcolemmal damage for specific fiber types generated by direct comparisons of the red and green channels (Figure 1C,D), revealed no differences between high and low responders in terms of the proportion of each fiber type exhibiting sarcolemmal damage (Table 2).

**Table 2:** Percentage fiber type and the proportion within each fiber type category that were damaged fibers (loss of dystrophin continuity) comparing low responders and high responders.

	Type I muscle fiber		Type II muscle fiber		Type IIa muscle fiber		Type IIx muscle fiber	
Responders	(%)	Damaged (%)	(%)	Damaged (%)	(%)	Damaged (%)	(%)	Damaged (%)
Low	35.2 ± 10.5	9.9 ± 8.3	64.8 ± 10.5	12.9 ± 5.7	59.8 ± 16.6	12.2 ± 5.4	8.8 ± 7.2	22.5 ± 18.2
High	37.6 ± 9.5	10.4 ± 6.1	62.4 ± 9.5	13.2 ± 7	52.2 ± 12.4	13.6 ± 8.2	10.2 ± 10	16.2 ± 10.7


*Data are presented as mean ± SD. No significant differences were observed between high and low responders in the percentage of muscle fiber type and in percentage of damaged muscle fibers at day 3 after the plyometric exercise intervention. The muscle biopsy was performed in the vastus lateralis.*

**FIGURE 2:**
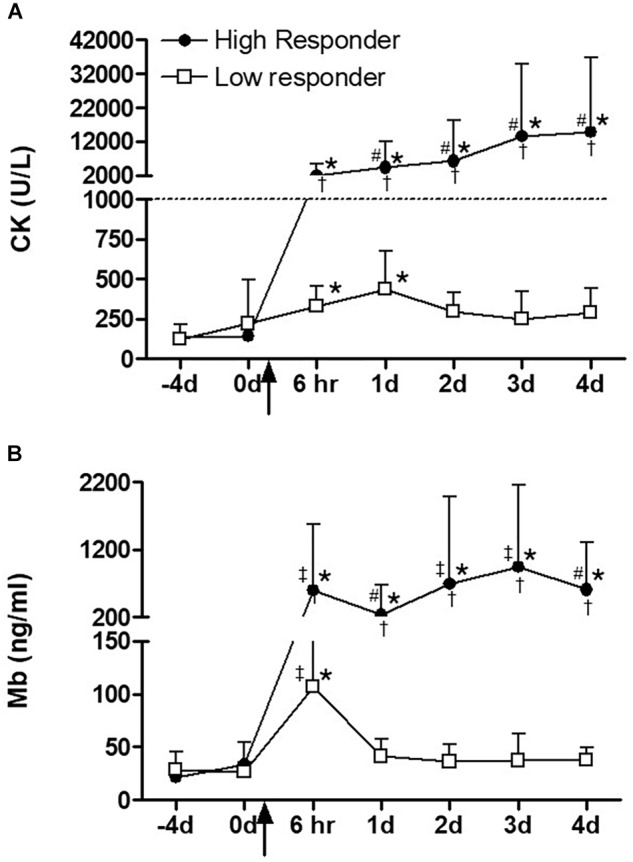
**(A)** Serum creatine kinase (CK) and **(B)** myoglobin (Mb) over time before and after a single bout of plyometric exercise. The square and circle dot indicate high (*n* = 10) and low responder (*n* = 16) groups, respectively. The dashed line indicates the exertional rhabdomyolysis cut-off used in this study. The arrow indicates the plyometric exercise intervention. ^∗^Significantly different from time points –4 and 0 day, (*P* < 0.001), ^#^significantly different from time point 6 h, (*P* < 0.05), ^‡^significantly different from time point 1 day, (*P* < 0.01), ^†^significantly different response between high and low responders, (*P* < 0.0001). Data are expressed as mean ± SD.

In terms of indirect indicators of muscle damage, serum CK activity was significantly elevated from baseline and pre-exercise values at both 6 h (*P* < 0.00001) and day 1 (*P* < 0.00001) after plyometric exercise intervention in the low responders, after which it returned toward baseline. In the high responders, a greater magnitude of increase (ANOVA effect of group, *P* < 0.0001) was observed for CK activity in response to the exercise intervention. In this group, CK activity remained at significantly higher levels when compared to pre-exercise values for all time points assessed (Figure 2A). A similar pattern to CK was observed for serum Mb, with greater and more sustained response in the high responders (Figure 2B). The only exception was that for Mb, the values in low responders returned to baseline levels already at day 1.

### Inflammation

Total WBC and neutrophil counts for both high and low responders were significantly but transiently increased 6 h after exercise, following which it returned to baseline values (Table 2). Both WBC and neutrophil counts peaked at significantly higher levels in high responders than low responders at 6 h (*P* < 0.5).

**FIGURE 3:**
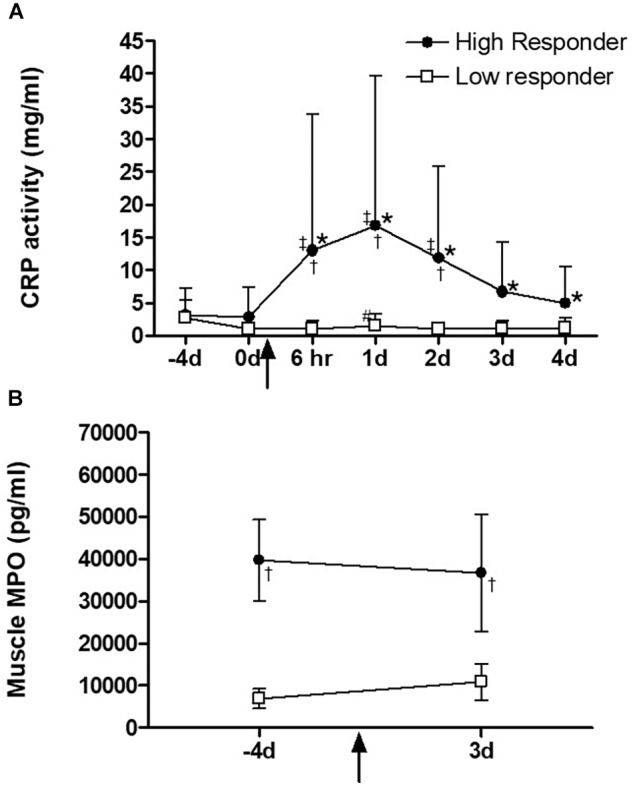
**(A)** Serum C-reactive-protein (CRP) and **(B)** skeletal muscle myeloperoxidase (MPO) levels before and on day 3 after exercise intervention. The square and circle dot in the lines indicate high (*n* = 10) and low (*n* = 16) responders, respectively. ^∗^Significantly different from time points –4 and 0 day, (*P* < 0.001), ^#^significantly different from time point 6 h, (*P* < 0.05), ^‡^significantly different from time point 1 day, (*P* < 0.01), ^†^significantly different response between high and low responders, (*P* < 0.0001). Data are expressed as mean ± SD.

Serum CRP levels increased (*P* < 0.05) transiently on day 1 after exercise in the low responders (Figure 3A). High responders had a more robust response, with significantly higher CRP levels, which were sustained at levels significantly higher than pre-exercise levels for all post-exercise time points. A significant group difference was evident from the 6 h time point to day 2. Pre-exercise intramuscular MPO levels were significantly higher (*P* < 0.05) in high responders when compared to low responders at baseline (Figure 3B). Neither group showed a significant MPO response to plyometric exercise at 3 days after plyometric exercise, which was the only time point after the intervention that a biopsy was taken.

**(A)** Serum C-reactive-protein (CRP) and **(B)** Myeloperoxidase (MPO) levels in skeletal muscle before and on day 3 after exercise intervention. The triangle and circle dot in the lines indicate high (*n* = 10) and low (*n* = 16) responders, respectively.

### Profile of MLCK Single Nucleotide Polymorphisms

All three alleles for MLCK C49T were observed among our participants: Twelve participants presented with the homozygous wild type CC allele, 6 with the CT allele and 7 with the TT allele. Participants with the CC allele had circulating CK and Mb activities of 7806 ± 5827 U/L and 299 ± 132 ng/ml, respectively, 3 days after plyometric jumping, versus somewhat lower values for those with the heterozygote allele (CT: 4485 ± 2912 U/L; Mb 227 ± 58 ng/ml) or the mutant homozygous allele (TT: CK 2442 ± 1779 U/L; Mb 146 ± 37 ng/ml).

## Discussion

To date, it remains unclear why, within a group of individuals exercising in a similar environment and at similar intensity, the development of exertional rhabdomyolysis is only observed within a small percentage of these individuals (Clarkson et al., 2005; Devaney et al., 2007; Yamin et al., 2008). The strength of the current study is that the unexplained dilemma of low and high responders to eccentric exercise was assessed from multiple points of view with the same volunteers within the same study. These viewpoints ranged from perceived pain, which was not similar at all time points between the two groups, to blood biomarkers of damage and inflammation, to genetic polymorphism testing (variants of MLCK gene) and muscular parameters. The results provide evidence suggesting that pre-existing elevated skeletal muscle MPO pre-disposes to higher CK responsiveness following unaccustomed plyometric exercise and that the inflammatory response, assessed here by subsequent CRP elevations in the days after muscle damaging exercise, is exacerbated in high CK responders. While other factors, such as muscle and genetic characteristics, are suggested to be contributing factors to muscle damage, these are not clearly evident unless a larger number of individuals are assessed and are therefore unlikely to be predictive in the individual setting.

In the literature, reports have found a poor correlation between perceived pain reporting and the degree of change in other indirect markers of muscle damage. For example, pain was found to be elevated 1 day post-marathon and while pain resolved, CK levels remained elevated 2 days after (Clifford et al., 2017). Furthermore, CK activity does not correlate well with the amount of structural damage or the reduction of muscle function and thus is an unreliable marker (Sorichter et al., 1997; Chimera et al., 2004). Nosaka et al. (2002) who used an eccentric elbow flexor model of muscle damage, reported that delayed onset muscle soreness (DOMS) correlated poorly not only with eccentric exercise-induced muscle damage and loss of maximal isometric force, but also smaller elbow joint angles, larger upper arm circumference and plasma CK activity. In the current study, despite all participants completing the required number of jumps between 95 and 100% of maximal jump height, the low CK responders reported significantly more perceived pain at the early time point of 6 h after plyometric jumping than the high responder group. The high responder group reported significantly higher delayed perceived soreness scores compared to the low responder group on days 3 and 4 following the exercise intervention, in particular when soreness was assessed in the squat position. This suggests that soreness should be assessed when the affected muscle groups are under eccentric strain, even if this is static. Coaches and athletic trainers should be aware that delayed soreness scores should be assessed for at least 4 days or more following intensive plyometric training in order to distinguish individuals possibly at risk of exertional rhabdomyolysis.

Muscle ultrastructural (*Z*-disk streaming) and structural damage (loss of dystrophin stain) was evident using electron and fluorescent microscopy, respectively, 3 days after the plyometric intervention. Nosaka et al. (2001) have reported that the repeated bout effect may last up to 6 months following an initial heavy eccentric exercise bout. However, some volunteers with a moderate to extreme response to unaccustomed eccentric exercise, who performed a second bout of eccentric exercise 3 weeks after the first bout, exhibited deformed fibers after the second bout (Paulsen et al., 2013). Given the qualitative evidence of muscle damage after the acute eccentric exercise intervention in all participants in the current study, it is unlikely that any of the participants did not fulfill the inclusion criterion of no habitual or recent training or plyometric exercise of the lower body. In previous studies, we observed acute bouts of plyometric jumping resulted in preferential fast twitch fiber damage (Macaluso et al., 2012b, 2014). McEwen and Hulland (1986) also observed preferential degeneration, but not exclusively to type II fibers, in horses affected by exertional rhabdomyolysis. Together, these findings could suggest that individuals with a greater percentage of fast twitch fibers may present more fiber damage, resulting in higher CK levels. However, no significant difference was observed between high and low responders in the proportions of fast and slow muscle fibers in the current study. The fiber type distribution was variable in both groups, which may explain this finding. A limitation is that with the size of the current study, there were no subjects with a very high proportion of fast twitch fibers. Therefore, it cannot be excluded that an even higher proportion of fast twitch fibers than that observed in the participants of the current study may be predictive of exertional rhabdomyolysis risk in other individuals.

Exertional rhabdomyolysis is common amongst military recruits (Hill et al., 2017), especially in the early phases after entry into service and if unaccustomed, excessive training is undertaken. The volume of such training is typically higher than that in laboratory research studies and the cut-off point for rhabdomyolysis may be as high as 5 times the upper limit of the normal, typically in the range of 1300 U/L (Warren et al., 2002). However, since signs such as dark colored urine are part of the diagnostic criteria for definitive clinical diagnosis of rhabdomyolysis (Hill et al., 2017), it has been found that exertional rhabdomyolysis may be underreported in some settings (Sauers et al., 2016). Therefore, in the current study a slightly lower cut-off limit of 1000 U/L was used, which is similar to other laboratory studies (Lee and Clarkson, 2003; Thoenes, 2010).

Recent reports suggest that SNPs may be responsible for inter-subject damage variability and CK response after eccentric exercise (Scalco et al., 2016). These studies have already established the genotypes of the different SNPs using larger study populations. Clarkson et al. (2005) demonstrated that individuals homozygous for the rare MLCK 49T allele (4% of 152 participants) had significantly greater increases in CK and Mb compared to the heterozygotes. The current data was not in line with this previous publication. Five of the seven individuals with this genotype were low responders for CK release and all of them were low responders for Mb. A major difference between the current cohort and that of Clarkson et al. (2005) is that 28% of the small cohort actually presented with the TT-homozygous allele. Therefore, this allele cannot be considered rare in our population. Although it might be considered a limitation for genotype assessment that the number of subjects in the current study was relatively low, taking the previous results and the current results together, they indicate the necessity of conducting genotype-phenotype studies in multiple populations around the world.

The two most interesting results of this study were the differences between the groups in the CRP profile and the baseline intramuscular MPO activity. High responders presented with significantly higher CRP levels following eccentric exercise when compared to their low responder counterparts, despite similar levels at baseline. Indeed, CRP was higher in high CK responders already at 6 h post-exercise and continued to be higher on days 1 and 2 when compared to the low responders, indicating prolonged cross-talk between damaged muscle and the liver. No group differences were evident at days 3 and 4, although high responders were still elevated from their own baseline at these two later time points. Although circulating neutrophil levels were significantly greater in the high responder group at 6 h post-exercise compared to the low responder group post-exercise, they did not remain elevated in the days following the intervention. Within 2 h after mechanical damage, neutrophils accumulate in the injured area (Hertel, 1997) and phagocytose cellular debris via the production of oxygen free radicals (Hertel, 1997; Toumi and Best, 2003; Pizza et al., 2005). Neutrophil activation may cause damage to cell membranes and surrounding non-injured tissue by rapidly releasing high concentrations of ROS and oxygen free radicals through a respiratory burst (Hertel, 1997; Brickson et al., 2001). In the context of unaccustomed eccentric exercise, this would be considered as secondary damage. By using a single stretch injury model in rabbit tibialis anterior muscle, Brickson et al. (2003) demonstrated that muscle fiber damage could be reduced by inhibiting neutrophil respiratory burst with a monoclonal antibody (M1/70). Furthermore, this model also showed the preservation of muscle structural proteins desmin and dystrophin (Brickson et al., 2003).

Myeloperoxidase is primarily expressed by neutrophils and plays an important role in microbicidal activity and cell debris destruction within the phagosome via oxidizing reactions (Hurst, 2012). Circulating monocytes also elevate intracellular MPO in response to eccentrically biased exercise such as downhill running (van de Vyver et al., 2016). Previous reports indicate that muscle loading during exhaustive exercise results in increased MPO activity per neutrophil (Suzuki et al., 1996) and downhill running increases intramuscular MPO (van de Vyver and Myburgh, 2014). Notably, the current data demonstrated higher MPO at baseline within the tissue targeted by the exercise in the high responders even before the plyometric intervention, which remained high at 3d after. This suggests that an inability to resolve MPO in muscle may contribute to a relatively exacerbated CRP response independent of the magnitude of the acute neutrophil response. It is unclear if the intramuscular MPO observed was of neutrophilic or monocytic origin. Given that there was no evidence of overt muscle pathology in the baseline biopsy samples, it is possible that pro-inflammatory macrophages may have been basally active in the high responder group’s muscle. However, MPO has also been shown to delay neutrophil apoptosis in an *ex vivo* model and to delay resolution of acute lung injury in mice (El Kebir et al., 2008). Neutrophil quantification in the muscle samples for future studies will be important. Not only in muscle fibers, but in the interstitial spaces between fibers; in the perivascular areas and beneath the epimysium in order to explain the difference in baseline MPO levels between high and low responders (Malm et al., 2004). In the current age of exercise prescription for rehabilitation and in groups with underlying disease, knowledge of baseline intramuscular MPO status may be valuable for decisions regarding the type of training prescribed. Merritt et al. (2013) showed that aged populations are particularly susceptible to muscle inflammation. This study also provided evidence that the skeletal muscle regenerative response is impaired with age following unaccustomed eccentric exercise, due to either high pro-inflammatory signaling at rest or a greater inflammatory response to damage and subsequent myoblast impairment.

In conclusion, in relatively healthy individuals, CRP responses to training are recommended for monitoring purposes and may indicate that a more conservative approach to training prescription should be followed where exercise interventions may be used as therapy for an individual’s recovery and in aged populations. In aged individuals and other individuals with conditions that may already put them at risk for compromised intramuscular inflammation, response to eccentric contraction and perceived pain should be assessed under static eccentric conditions.

## Author Contributions

AI, FM, and KM performed the experiments. AI, FM, and CS conceived the project and analyzed the data. All authors contributed to critical analysis. AI, FM, CS, and KM wrote the manuscript and all authors approved the final manuscript for publication.

## Conflict of Interest Statement

The authors declare that the research was conducted in the absence of any commercial or financial relationships that could be construed as a potential conflict of interest.
